# Informed decision-making in prioritising product variants

**DOI:** 10.7717/peerj-cs.2778

**Published:** 2025-06-30

**Authors:** Diana Borrego, Ángel Jesús Varela-Vaca, María Teresa Gómez-López, Rafael M. Gasca

**Affiliations:** Departamento de Lenguajes y Sistemas Informáticos, Universidad de Sevilla, Sevilla, Spain

**Keywords:** Feature models, Software product line, Informed decision-making support, Prioritisation

## Abstract

Feature models (FMs) play a crucial role in software product lines (SPLs) by representing variability and enabling the generation of diverse product configurations. However, the vast number of possible configurations often makes it challenging to identify the most suitable variant, especially when multiple criteria must be considered. Multi-criteria decision-making (MCDM) methods, such as analytic hierarchy process (AHP), technique for order of preference by similarity to ideal solution (TOPSIS), and VIseKriterijumska Optimizacija I Kompromisno Resenje (“multicriteria optimization and compromise solution”) (VIKOR), are effective for ranking configurations based on user-defined preferences. However, the application of disparate MCDM techniques to the same feature model with identical criteria can yield conflicting rankings, thereby complicating the decision-making process. To address this issue, we propose a novel framework that systematically integrates multiple MCDM methods to prioritise product configurations and provides informed decision support to reconcile ranking discrepancies. The framework automates the prioritisation process and offers a structured approach to explain differences between rankings, enhancing transparency and user confidence in the final selection. The framework’s effectiveness has been validated through real-world case studies, demonstrating its ability to streamline configuration prioritisation and support consistent, preference-driven decision-making in complex SPL environments.

## Introduction

In today’s software industry, variability models are used to compactly represent all product variants of a software product line (SPL) in terms of features (Benavides, Segura & Cortés, 2010). An SPL integrates similar software products with features in common, but not all of them. Feature models (FM) are a type of variability model that allows compacting of the variability representation of a family of products (Metzger & Pohl, 2014). The use of FMs is widespread and several studies and tools support their management and analysis (Horcas, Pinto & Fuentes, 2023; Varela-Vaca et al., 2019). One of the main functionalities of these tools is their capacity for automatic reasoning and the generation of a set of partial or complete configurations from a specified model.

A product variant can be represented by a product configuration, which includes the selection of features for an FM. The vast number of possible product configurations poses a significant challenge: how to efficiently identify and prioritise the configurations that best meet user requirements (Fernández-Amorós et al., 2024). While FMs provide a compact representation of these configurations, the sheer volume of potential options can overwhelm decision-makers. This complexity highlights the need for systematic methods that can assist in ranking configurations based on multiple criteria, ensuring that the most suitable product variants are selected. For example, a car manufacturer’s product line (Astesana, Cosserat & Fargier, 2010) can potentially include an exponential number of configurations, influenced by features such as colour, engine type, Bluetooth capability, and navigation technology. However, it should be noted that not all configurations are supported, for example, the inclusion of the navigation system but not that of Bluetooth. In addition, it may be unfair to present all possible configurations and analysing them would be pointless, time consuming and costly. It would be better to present a subset of configurations that meet the user’s needs, for example those that prioritise fuel economy over boot space. Consequently, the prioritisation of a subset of configurations based on user-defined criteria becomes imperative to facilitate streamlined decision-making processes.

Designing a specific product within a product line involves optimising the selection of features to identify the ‘best’ product. Product configuration prioritisation offers significant benefits, such as efficiency in decision-making by streamlining the selection process, optimisation of resources by focusing on high-potential options, and reduction of uncertainty by avoiding less promising configurations. It also enables strategic alignment with organisational goals, continuous improvement by identifying areas for improvement, and effective risk management by proactively addressing configurations with significant risks. Clear rankings also facilitate communication and collaboration, contributing to quality improvement in various areas such as software development, engineering, research, and design.

Determining the best set of features becomes a multi-objective optimisation problem (Henard et al., 2015). Multi-criteria decision-making (MCDM) (Ishizaka & Nemery, 2013) is a mature field that has been applied in several contexts and can be applied in this context of product configuration. In Saber, Bendechache & Ventresque (2021), the multi-objective problem was analysed by incorporating MDCM into SPL. However, different ordered lists can be obtained for the same feature model and criteria, depending on the MCDM applied. This variation gives rise to a critical challenge: the process of resolving discrepancies in the rankings produced by different MCDM methods and determining which configuration best suits the user’s preferences. There is currently no systematic framework that integrates multiple MCDM methods to prioritise configurations derived from feature models.

The need to merge the results obtained by MCDMs is a problem that does not only exist in the context of SPL (Egan, 2024). For example, in companies and organisations, it is often difficult to bring together the prioritisation criteria of all stakeholders in decision-making meetings, which can lead to low productivity or even failure to produce the best products. The main research questions to be addressed are *What is the best decision if various MCDM methods obtain different rankings, and how do the specific features of the FM play a role in the decision?*

To address these challenges, this article proposes a novel framework that systematically integrates multiple MCDM methods—analytic hierarchy process (AHP) (Saaty, 1990), technique for order of preference by similarity to ideal solution (TOPSIS) (Hwang & Yoon, 1981), and multicriteria optimization and compromise solution (VIKOR) (Opricovic & Tzeng, 2007)—for the prioritisation of configurations derived from feature models. The framework not only automates the prioritisation process but also provides a methodical approach to resolving discrepancies between the rankings generated by different MCDM techniques. By offering informed decision support, the framework enhances transparency, improves trust in the selection process, and facilitates reproducibility.

The integration of multiple MCDM methods into FM prioritisation addresses a critical gap in the field of SPLs. By systematically reconciling discrepancies between different ranking methods, this approach enhances decision-making transparency and reliability. Beyond SPLs, the proposed framework has the potential to be applied in various domains where complex configurations and multi-criteria decisions are required, such as cloud service selection, IoT system configuration, and embedded systems design. From a pragmatic standpoint, the automation of the prioritisation process has the potential to reduce the time and effort required from experts, streamline product development workflows, and ensure that configurations are aligned with user-defined priorities. This, in turn, leads to more efficient resource allocation, better product quality, and increased confidence in decision-making processes across diverse software and engineering environments.

**Figure 1:**
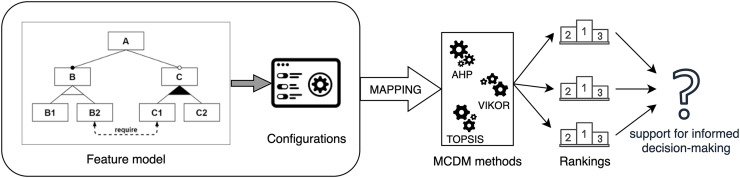
Overview of the contribution application process.

The general idea of this article is represented in Fig. 1, and the main contributions are:

- A novel framework that automates the prioritisation of configurations obtained from feature models by integrating multiple MCDM methods.
- A systematic approach for analysing and resolving ranking discrepancies, providing informed decision support to guide users in selecting the most appropriate configuration.
- An evaluation of the framework through real-world case studies, with a view to demonstrating its applicability, robustness, and scalability in handling complex feature models.

This article is divided into the following sections. “Foundations” introduces the concept of Feature Model and presents some decision-making techniques. “Proposed methodology for applying MCDM methods” introduces the proposed methodology for the application of MCDM methods and presents the different phases of the process. “Detailed Application of AHP, TOPSIS, and VIKOR” provides a detailed explanation of the configuration and application phases for AHP, TOPSIS, and VIKOR. “Informed Decisions: In-Depth Examination of Ranking Discrepancies” details the phase focused on supporting informed decision-making when discrepancies are observed between the rankings obtained by the different techniques. The whole process is applied to a real example and is outlined in “Results”. “Discussion” discusses the implications, strengths, and limitations of the proposed approach, including its scalability, reliance on expert input, and potential enhancements through automated techniques or alternative decision-making frameworks. “Related Work” analyses related works in the literature. Finally, “Conclusions and Future Work” concludes the article and presents ideas for future work.

## Foundations

This section briefly introduces FMs and their use to represent SPLs. Likewise, some MCDM methods are briefly presented.

### Feature models

**Figure 2:**
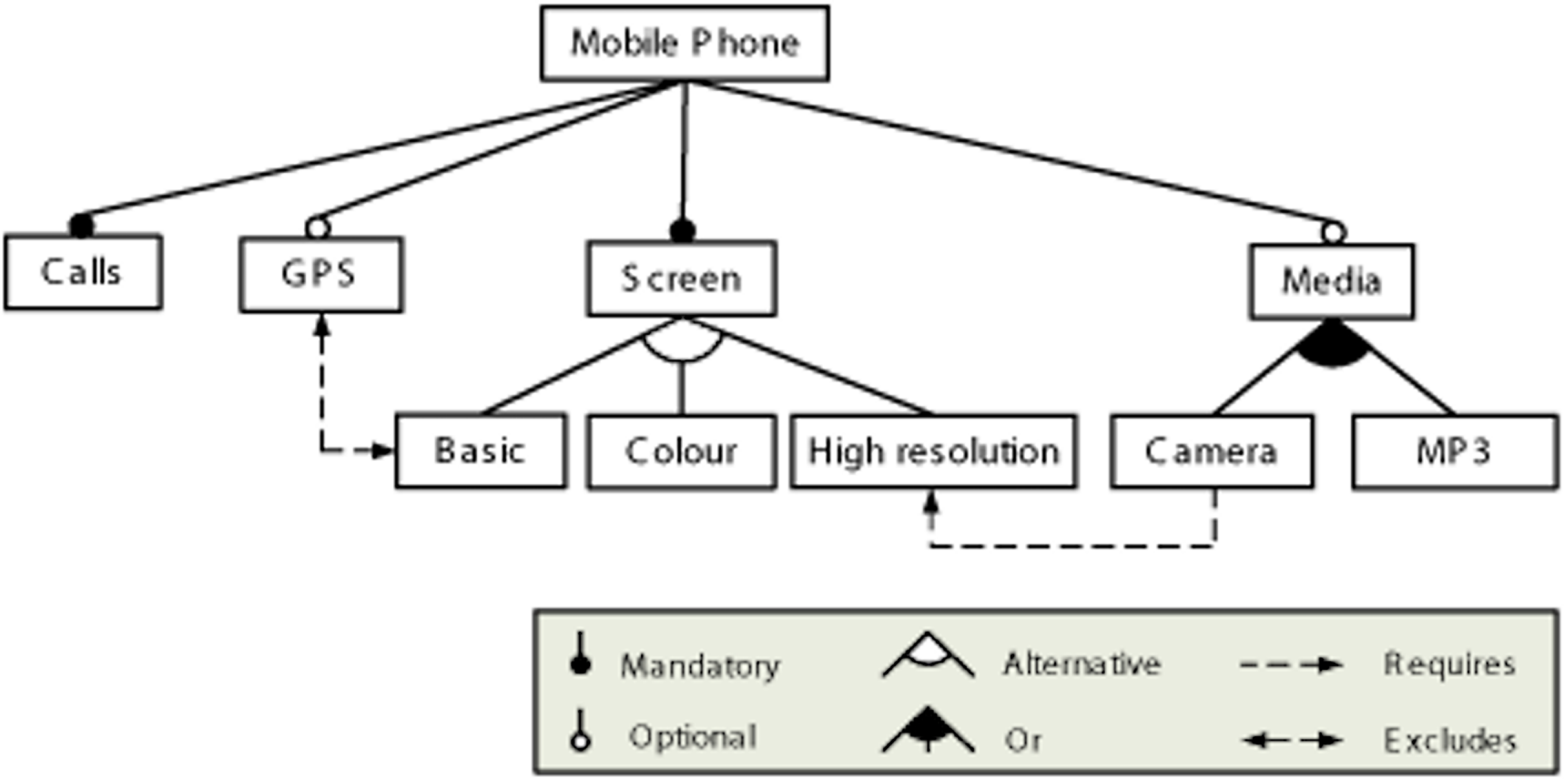
Example of feature model.

SPLs (Clements, 2001; Becker et al., 2001) represent a set of products that share common features, with the objective of facilitating the creation of products based on user requirements. They can be described using models, where one of the main notations for expressing these models is the Feature Model (FM) notation (Schobbens et al., 2007), which describes the set of properties in an SPL in terms of features and the relationships between them. A FM represents the features of a product and the configuration options for each of them in tree form. An example of how FMs are usually represented is shown in Fig. 2.

Features are related through dependencies; these can be mandatory, optional, group cardinality, inclusion, or exclusion. Each of these is explained below:

- **Mandatory.** When this relationship is established between two features (child and parent respectively) of a model, the child feature must be selected when the parent feature is selected in a configuration. In the example, it is used to relate the features *Mobile Phone* and *Calls*.
- **Optional.** This relationship is established between parent and child features and indicates that the occurrence of the parent feature in a configuration does not imply that the child feature must also occur. In the example feature model, it is used to relate the *Mobile Phone* and *GPS* features.
- **Or-relationship.** When this relationship is established between a parent feature and its set of child features, when the parent feature is selected in a configuration, one or more of its children must be selected. In the example feature model, it is used to relate feature *Media* and its configuration options *Camera* and *MP3*.
- **Alternative.** When this relationship is established between a parent feature and its set of child features, when the parent feature is selected in a configuration, one and only one of its children must be selected. In the example feature model, it is used to relate feature *Screen* and its configuration options *Basic*, *Colour* and *High Resolution*.
- **Requires.** If a feature X requires another feature Y, then when X is selected in a configuration, Y must also be selected. In the example feature model, it is used to restrict features *Camera* and *High Resolution*.
- **Excludes.** If a feature X excludes another feature Y, then when X is selected in a configuration, Y cannot be selected. In the example feature model, it is used to restrict features *GPS* and *Basic*.

A concrete product variant of the feature model is derived through a configuration process: binding the variation points and instantiating the different features of the model. For the feature mode above, some of the possible configurations of a mobile phone could be {Calls, Screen, Basic, GPS}, {Calls, Screen, Colour, Media, Camera}, and so on. As mentioned previously, these configurations could be sorted according to certain criteria.

### Multi-criteria decision-making methods

The configurations derived from a feature model could be ranked according to certain criteria that capture the characteristics (factors, properties, or attributes) of a decision problem from multiple aspects. In these problems, alternatives are considered to be available choices. These alternatives can be processed by multi-criteria models for selection or ranking purposes. An attribute is a characteristic of an alternative that can lead us to think about the ‘pros’ and ‘cons’ of the different alternatives.

MCDM methods can be classified in several ways. First, based on the initial information used, these methods can focus on a set of specific attributes—referred to as multi-attribute decision-making (MADM)—or address multiple objectives that may conflict—known as multi-objective decision-making (MODM). Secondly, they can be categorised according to the type of initial information considered, which can be deterministic, stochastic, or uncertain. Finally, MCDM methods can be classified according to the number of decision groups involved, distinguishing between single and multiple groups.

Depending on the strategy used to rank the alternatives, there are different methods: distance-based methods (*e.g*., TOPSIS and VIKOR); pairwise comparison methods (*e.g*., AHP and ANP); scoring methods (*e.g*., SAW, Weighted Sum Model (WSM), Weighted Product Model (WPM), *etc*.); and outranking methods (*e.g*., PROMETHEE, ELECTRE).

In the context of complex decisions, the MCDM methods used for our proposal are AHP, TOPSIS, and VIKOR. Although other MCDM methods could be included in our framework, these methods have been chosen because of their wide acceptance and use in the academic and business communities, and because of their effectiveness in dealing with discrete problems involving multiple criteria and alternatives. Their popularity stems from their ability to address complex decision issues, their versatility, and their strong theoretical foundations resulting from decades of research and successful applications. The following subsections introduce these MCDM methods.

#### AHP

AHP (Saaty, 1990) (an acronym for “Analytic Hierarchy Process”) is a structured technique for organising and analysing complex decisions developed by Thomas L. Saaty in the 1970s. The basis of the AHP is the quantification of the weights of the decision criteria when more than one influences the decision-making process. Its purpose is not to identify the *right* decision but to help rank the alternatives from the best to the worst for a given objective, providing a framework for structuring a decision problem, representing and quantifying its elements, relating the elements to the objectives pursued and evaluating the different solution alternatives.

According to Saaty (1990), the AHP method involves three main steps: (1) Decomposition: arrange criteria and alternatives in a hierarchical structure; (2) comparative judgment: perform pairwise comparisons to evaluate elements based on their influence; (3) synthesis: calculate weights and priorities from comparison matrices.

In AHP, pairwise comparison matrices are fundamental for evaluating preferences and relative importance relationships between criteria and alternatives. These matrices allow for systematic and consistent comparisons between criteria, subcriteria, and alternatives.

AHP can still be applied when not all criteria are involved in each alternative by adapting the methodology. In these cases, comparisons are made between specific values of the criteria, rather than comparing alternatives directly. This approach, known as “Pairwise Comparison of Attributes” or “Pairwise Comparison of Criteria” (Harker, 1987), allows relative priorities to be obtained for each value, which are then used to calculate the final prioritisation of alternatives as a weighted sum of their component values.

For configurations obtained by an FM, AHP offers the advantage of systematically evaluating multiple criteria and their interrelationships, facilitating the decomposition of complex decision problems into more manageable parts.

As part of the methodology, the comparison matrix is iteratively multiplied by itself until no significant difference is observed, ensuring convergence to a stable and robust solution. Furthermore, the consistency index is used to assess the reliability of the comparisons, and in our case, we will only consider the indices below 10%, to ensure consistent and valid results.

Classical AHP is based on the clear judgements of decision-makers, but is not able to reflect vague human thoughts or the uncertainty of information. This could be solved by a fuzzy AHP. In addition, AHP does not take into account the interrelationships and feedback between criteria.

#### TOPSIS

TOPSIS (an acronym for “Technique for Order of Preference by Similarity to Ideal Solution”) is a method originally developed by Hwang & Yoon (1981), and later developed by Yoon (1987) and Hwang, Lai & Liu (1993). Over the last decade, TOPSIS has been used to make decisions in areas such as supply chain management, the environment, energy, health or economics, to classify or select different alternatives or to optimise processes (Palczewski & Sałabun, 2019; do Carmo Silva, Gomes & da Costa Junior, 2019; Shih & Olson, 2022).

It is based on the concept that, given a set of alternatives and two ideal solutions—a positive (PIS) and a negative (NIS)—the best alternative is the one closest to the positive ideal solution and farthest from the negative ideal solution.

In this method, the decision matrix is first normalised according to profit and cost criteria, and then weighted according to the importance of each criterion. From this, the PIS and the NIS are calculated, representing the best and worst possible values for each criterion. The alternatives are then evaluated by calculating their distance from the PIS and NIS, and finally a score is assigned to each alternative based on these distances, providing a ranking of the options.

#### VIKOR

Like TOPSIS, VIKOR is based on distance metrics. VIKOR (acronym for “VIseKriterijumska Optimizacija I Kompromisno Resenje”) is a methodology, originally developed by Opricovic (1998) as part of his doctoral thesis in the late 1970s to solve decision problems with conflicting and non-comparable criteria.

Since the mid-2000s, VIKOR has become a multi-criteria decision-making methodology that has attracted the interest of many researchers around the world. According to the study by Mardani et al. (2016), VIKOR has been used in fields such as operations research, management science, sustainability, and renewable energy.

VIKOR has the advantage of providing a ranking based on proximity to the best and worst case solutions, as well as the geometric mean, allowing different scenarios to be considered when selecting optimal configurations. Its advantage lies in its application to situations where a balance between optimisation and robustness is sought, offering a compromise solution that minimises the potential loss in case of unfavourable variations in the criteria.

In this method, given a set of alternatives, criteria and a value $v \in [0,1]$ to be used in calculating the Q index, the process begins by identifying the best and worst case solutions based on the optimal and least optimal values for each criterion. The S and R indices are then calculated, where S represents how well an alternative meets the criteria and R measures how much it falls short according to the worst case scenario. The Q index is calculated by combining the S and R indices with the value v, giving a single value for each alternative. Finally, the alternatives are ranked according to their Q Index, with lower values indicating higher priority. The resulting ranking represents a compromise solution that balances group utility with minimising individual regret.

Opricovic & Tzeng (2007) presents a comparative analysis of VIKOR with other methods, such as TOPSIS and outranking methods, by discussing their characteristics and their application results.

## Proposed methodology for applying MCDM methods

**Figure 3:**
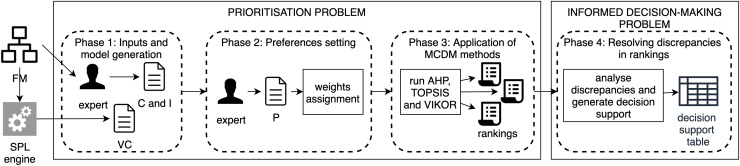
Methodology for the application of prioritisation techniques.

To formalise the configuration prioritisation process, we define a prioritisation problem and an informed decision-making problem. The former is the task of obtaining the configuration rankings by applying MCDM methods, while the latter is the task of interpreting the discrepancies in the rankings to help the expert make a comprehensible decision. For the application of MCDM methods in the context of FM, we propose the methodology described in Fig. 3. This process consists of the following four phases:

(1) **Phase 1: Inputs and model generation.** From the feature model, (1) the expert determines the features to be considered as criteria and instances in the prioritisation process, and (2) all valid configurations are obtained (using an external tool). All these elements are inputs to our prioritisation process.
(2) **Phase 2: Preference setting.** Based on the features identified as criteria in the previous phase, the expert introduces the pairwise information that will determine the most relevant features.
(3) **Phase 3: Application of MCDM methods.** In this phase, MCDM methods are applied, in our case study these are AHP, TOPSIS and VIKOR.
(4) **Phase 4: Resolving discrepancies in the rankings.** This phase is responsible for helping the user to interpret the possible differences between the ranks obtained.

To make the proposal clearer and more formal, the following subsections explain each phase in detail as applied to a particular FM.

### Phase 1. Inputs and model generation

As mentioned above, the parameters needed to start the process are the FM, a selection of features to be used as criteria in the prioritisation process, and the set of valid configurations according to the FM.

#### FM

**Figure 4:**
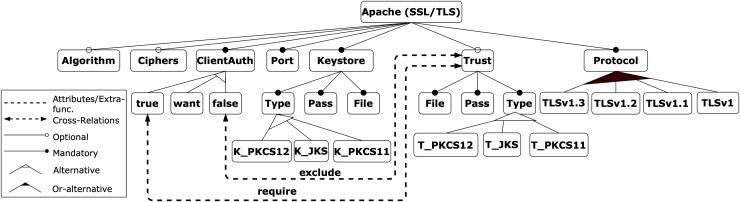
Apache feature model.

To facilitate the explanation of the proposal, we use an illustrative example related to the field of cybersecurity, taken from the contribution in Varela-Vaca et al. (2019). It is based on the security feature model of an Apache Tomcat server, which represents the attributes required to configure an HTTP connector with SSL support, as shown in Fig. 4. The model consists of 27 features, 10 mandatory relations, three optional relations, one or-alternative relations, three alternative relations, one require and one exclude constraints.

#### Features involved in the prioritisation

In the context of MCDM, the expert plays a crucial role in mapping the feature model to the specific configurations and criteria. Although a configuration may contain many features, not all of them need to be involved in the prioritisation process, only those identified by the expert. Thus, the mapping process starts with the expert’s selection of the features to be considered as prioritisation criteria. This step discriminates the features that influence whether a configuration is considered better or worse concerning the defined prioritisation objective. Similarly, it must be determined which features (leaves of the model) are possible values that each criterion can take, *i.e*., its instances.

• Let *C* be the subset of internal FM nodes (features) considered as criteria for evaluating and comparing alternatives.
(1) $$C \subset F$$
For the Apache example, the criteria identified by the expert are ClientAuth, KeyStore, Trust, and Protocol features.
• Let *I* be the subset of FM leaf nodes (features) that the expert considers to be instances of *C*, *i.e*., the values that the criteria can take.
(2) $$I \subset F$$
For the Apache example, the enabled instances of each criterion are:
  – *ClientAuth*: true, want, or false.
  – *KeyStore*: JKS, PKCS12, or PKCS11.
  – *Trust*: JKS, PKCS12, or PKCS11.
  – *Protocol*: v1, v1.1, v1.2, and/or v1.3.

#### Valid configurations

Several configurations can be obtained, but not all of them are valid. That is, some configurations do not satisfy the constraints and relationships imposed by the model. Therefore, only valid configurations need to be considered. A valid configuration is a selection of features that satisfy all constraints and conditions in the FM.

• Let **VC** be all valid configurations for the given FM. Each configuration $vc \in VC$ is characterised by the selection of a set of features $f$, where $f \subset F$.

For the Apache example and using CyberSPL (Varela-Vaca et al., 2019), the possible configurations of the feature model are $576$. Among all of them, a subset of 10 configurations of *VC* is shown in Table 1^1^ 1Table 1 has been obtained through a practical implementation, in the AMADEUS framework (Varela-Vaca et al., 2020). AMADEUS is a framework solution that enables and supports the automatic analysis of security vulnerabilities in system configurations based on feature models. AMADEUS can automatically analyse the organisation’s infrastructure and identify vulnerabilities by querying vulnerability repositories.. These 10 are used as illustrative examples, but others could be chosen. However, as will be shown later in the application of decision-making techniques, the number of configurations to be prioritised does not significantly affect the performance of the prioritisation process.

**Table 1:** Selected configurations for Apache.

Config. No.	Apache product configuration
1	Apache–Algorithm–Ciphers–ClientAuth–true–Port–KeyStore–K_Type–K_JKS–K_Pass–K_File–Trust–T_File–T_Pass–T_Type–T_PKCS11–Protocol–TLSv1_1
2	Apache–Algorithm–ClientAuth–true–Port–KeyStore–K_Type–K_PKCS11–K_Pass–K_File–Trust–T_File–T_Pass–T_Type–T_PKCS12–Protocol–TLSv1_3
3	Apache–Ciphers–ClientAuth–true–Port–KeyStore–K_Type–K_PKCS12–K_Pass–K_File–Trust–T_File–T_Pass–T_Type–T_JKS–Protocol–TLSv1_2
4	Apache–Algorithm–Ciphers–ClientAuth–want–Port–KeyStore–K_Type–K_PKCS11–K_Pass–K_File–Trust–T_File–T_Pass–T_Type–T_PKCS12–Protocol–TLSv1_2
5	Apache–Algorithm–ClientAuth–want–Port–KeyStore–K_Type–K_PKCS12–K_Pass–K_File–Trust–T_File–T_Pass–T_Type–T_JKS–Protocol–TLSv1_1
6	Apache–Ciphers–ClientAuth–want–Port–KeyStore–K_Type–K_PKCS12–K_Pass–K_File–Trust–T_File–T_Pass–T_Type–T_PKCS11–Protocol–TLSv1_2–TLSv1_3
7	Apache–Algorithm–Ciphers–ClientAuth–false–Port–KeyStore–K_Type–K_JKS–K_Pass–K_File–Protocol–TLSv1_2
8	Apache–Algorithm–ClientAuth–false–Port–KeyStore–K_Type–K_PKCS12–K_Pass–K_File–Protocol–TLSv1_3
9	Apache–Ciphers–ClientAuth–false–Port–KeyStore–K_Type–K_PKCS11–K_Pass–K_File–Protocol–TLSv1_1
10	Apache–Algorithm–Ciphers–ClientAuth–want–Port–KeyStore–K_Type–K_PKCS12–K_Pass–K_File–Protocol–TLSv1_3

### Phase 2. Preference setting

Once the expert has decided which features are to be considered as criteria (*C*) and instances (*I*), comparisons between them, called preferences, are determined at this stage. Both ways of expressing preferences—through pairwise comparison matrices or numerical scales—involve a comparison between possible instances of each criterion to indicate which features are preferred over others in a configuration. To do this, the expert must establish the preferences to prioritise the valid configurations.

• Let *P* be the preferences about *C* and *I*.

These preferences *P* are assumed to be the same for all $n$ different MCDM methods, thereby ensuring the comparability of the outcomes. However, it should be noted that each MCDM method requires preferences to be expressed in a particular manner. For example, AHP requires them in the form of pairwise comparison matrices of criteria and instances, while TOPSIS and VIKOR require them in the form of numerical scales. This necessitates the allocation of distinct weights to each method, based on the preferences provided by the expert. This is explained in more detail for the Apache example below in “AHP application methodology, TOPSIS application methodology and VIKOR application methodology”.

Notably, the FM may allow features to have multiple values *via* the OR operator, introducing additional complexity. To illustrate this, consider the Protocol feature in the Apache example, which can take on any of the following combinations: v1.0, v1.1, v1.2 and/or v1.3. In order to ascertain preferences, it is necessary to consider all possible combinations of instances. This results in 16 possibilities to weight in the Protocol for the Apache example, which can be tedious, so a solution is proposed. Experts can specify preferences for each instance independently and categorise how OR relationships should be considered, called OR-feature type, with four options: (1) average weight, (2) weight of the best-rated instance, (3) weight of the worst-rated instance, or (4) sum of the weights of the instances. This approach simplifies the evaluation process and facilitates decision-making.

### Phase 3. Application of MCDM methods

Once all the necessary elements involved in a prioritisation method $(FM,C,I,VC,P)$ have been defined, the MCDM methods are applied, which are described in detail in “Detailed Application of AHP, TOPSIS, and VIKOR”.

After solving the prioritisation problem, the outcome is $n$ rankings of the configurations in *VC*, one per each different MCDM method applied $R = \{ {R_{1}},{R_{2}}, \ldots ,{R_{n}}\}$. The configurations in these rankings are ordered according to their importance based on the preferences assigned to the criteria and instances.

In the case of the Apache example, the three MCDM methods obtain rankings of the 10 selected configurations, as illustrated in Table 2. The numbers in the AHP, TOPSIS, and VIKOR columns correspond to the numbers of Apache configurations in Table 1.

**Table 2:** Rankings of Apache configurations for each MCDM method.

Ranking	Apache Config. No.		
	AHP	TOPSIS	VIKOR
#1	2	2	2
#2	3	3	3
#3	4	4	4
#4	1	5	6
#5	5	1	1
#6	6	6	5
#7	10	10	10
#8	8	8	8
#9	7	7	7
#10	9	9	8

As can be seen, the three MCDM methods have produced rankings that are not equal to each other. Specifically, the configurations in positions #4, #5 and #6 of the rankings are not in the same order. Therefore, the solutions provided are not consistent between them. In the event that the user is required to select between these options, it is necessary to provide assistance in determining the optimal choice in our case and the rationale behind it, thereby enabling the user to make an informed decision.

### Phase 4. Resolving discrepancies in rankings

When the configuration rankings obtained by the MCDM methods are not consistent, it is useful to analyse the differences between them to help the user interpret the discrepancies and thus facilitate the choice of configurations to apply. To explain why these choices are different, some considerations are required (*e.g*., Table 2 for the Apache example).

- (FM, *VC*, *C*, *I*, *P*), as determined for the previous prioritisation problem.
- The set of rankings $R = \{ {R_{1}},{R_{2}}, \ldots ,{R_{n}}\}$, where each ${R_{i}}$ is an ordered list of $m$ valid configurations $\{ v{c_{1i}}, \ldots ,v{c_{mi}}\}$.

Phase 4 focuses on interpreting the discrepancies observed in the rankings generated by the MCDM methods. By analysing the structure and variability of the feature model (*e.g*., OR relationships, alternatives and required features) in conjunction with the rankings, this phase aims to provide the user with practical insights to facilitate understanding of the trade-offs and enable informed decision-making. The methodology ensures that users can address complex prioritisation scenarios with clarity and confidence (see “Informed Decisions: In-Depth Examination of Ranking Discrepancies”).

## Detailed application of AHP, TOPSIS, and VIKOR

In this section, we provide a detailed analysis of the methods used in Phases 2 and 3 to prioritise the valid configurations. Phase 2 focuses on setting preferences through pairwise comparison matrices, while Phase 3 details the application of each method to compute the final rankings. Each method is examined in its own subsection, where we outline the process, inputs, and resulting rankings.

### AHP application methodology

Building on the work carried out in Phase 1, where the problem was structured into a set of criteria, instances, and alternatives (*i.e*., valid configurations *VC*), Phase 2 begins by incorporating the preferences of the decision-maker. For the AHP method, these preferences are captured through pairwise comparison matrices, which are used to evaluate the relative importance of each criterion and its alternatives. In the matrices, a value greater than 1 indicates that the criterion or alternative in a given row is more important than the one in a given column. Conversely, values less than 1 indicate a lower level of importance. This process is illustrated in the Table 3, which contains the pairwise comparison matrices for the Apache Tomcat server and the previously selected criteria.

**Table 3:** Pairwise comparison matrices.

(a) Criteria pairwise comparison matrix				
	ClientAuth	KeyStore	Trust	Protocol
ClientAuth	–	4	1	2
KeyStore	1/4	–	1/3	1/2
Trust	1	3	–	2
Protocol	1/2	2	1/2	–

(b) ClientAuth instances			
	True	Want	False
True	–	2	4
Want	1/2	–	2
False	1/4	1/2	–

(c) KeyStore and trust instances			
	JKS	PKCS12	PKCS11
JKS	–	1/2	2
PKCS12	2	–	4
PKCS11	1/2	1/4	–

(d) Protocol instances				
	v1	v1.1	v1.2	v1.3
v1	–	1/2	1/4	1/6
v1.1	2	–	1/2	1/4
v1.2	4	2	–	1/2
v1.3	6	4	2	–

Despite the presence of OR relationships in certain features, the expert only compares individual values of the features. For example, the expert determines that the feature *Protocol*, in the form of an OR relation, can be treated as an “average” feature type. This means that the weights for a configuration with multiple values for this feature are calculated as the average of the weights of the individual values. In this example, configuration number six includes different protocol versions *v1.2 & v1.3* (see Table 1), and Table 4 is automatically generated to replace Table 3d, so as to include this new value *v1.2 & v1.3*, whose weights for comparison are calculated as the average of the weights of *v1.2* and *v1.3*.

**Table 4:** Protocol pairwise comparison matrix with multiple values.

	v1	v1.1	v1.2	v1.3	v1.2 & v1.3
v1	–	1/2	1/4	1/6	1/5
v1.1	2	–	1/2	1/4	1/3
v1.2	4	2	–	1/2	3/4
v1.3	6	4	2	–	3/2
v1.2 & v1.3	5	3	3/2	3/4	–

Once preferences have been established, Phase 3 uses the AHP method to calculate priorities and generate the final ranking. Priorities are determined through the following steps:

- Obtaining priorities of criteria instances: The AHP method is used to solve the eigenvalue and eigenvector problems for each pairwise comparison matrix, providing the relative priorities of each criterion.
- Weight assignment: The weights for the instances of each criterion are derived from the calculated priorities. For example, the weights assigned to the criteria and their instances in the Apache Tomcat server example are shown in Table 5.
- Final prioritization of alternatives: The final priorities of the configurations are computed by calculating the weighted sum of the component instances for each configuration. The resulting priorities for the Apache Tomcat configurations are shown in Table 6, and the final ranking is presented in Table 7.

**Table 5:** Assignment of weights to criteria and instances.

**(a) Criteria**	
ClientAuth	37.01%
KeyStore	9.99%
Trust	34.51%
Protocol	18.50%
**(b) ClientAuth instances**	
True	21.15%
Want	10.57%
False	5.29%
**(c) KeyStore instances**	
JKS	2.85%
PKCS12	5.71%
PKCS11	1.43%
**(d) Trust instances**	
JKS	9.86%
PKCS12	19.72%
PKCS11	4.93%
**(e) Protocol instances**	
v1	0.88%
v1.1	1.76%
v1.2	3.52%
v1.3	7.05%
v1.2 & v1.3	5.29%

**Table 6:** Calculation of the priority of each Apache feature model alternative.

Alternatives	Features	Priority
Apache Config. No. 1	True (21.15%) | JKS (2.85%) | PKCS11 (4.93%) | 1.1 (1.76%)	30.69%
Apache Config. No. 2	True (21.15%) | PKCS11 (1.43%) | PKCS12 (19.72%) | 1.3 (7.05%)	49.35%
Apache Config. No. 3	True (21.15%) | PKCS12 (5.71%) | JKS (9.86%) | 1.2 (3.52%)	40.24%
Apache Config. No. 4	Want (10.57%) | PKCS11 (1.43%) | PKCS12 (19.72%) | 1.2 (3.52%)	35.24%
Apache Config. No. 5	Want (10.57%) | PKCS12 (5.71%) | JKS (9.86%) | 1.1 (1.76%)	27.90%
Apache Config. No. 6	Want (10.57%) | PKCS12 (5.71%) | PKCS11 (4.93%) | v1.2 & 1.3 (5.29%)	27.50%
Apache Config. No. 7	False (5.29%) | JKS (2.85%) | - | 1.2 (3.52%)	11.66%
Apache Config. No. 8	False (5.29%) | PKCS12 (5.71%) | - | 1.3 (7.05%)	18.05%
Apache Config. No. 9	False (5.29%) | PKCS11 (1.43%) | - | 1.1 (1.76%)	8.48%
Apache Config. No. 10	Want (10.57%) | PKCS12 (5.71%) | - | 1.3 (7.05%)	23.33%

**Table 7:** Ranking of the alternatives for apache feature model obtained with AHP.

Alternatives	Priority	Ranking
Apache Config. No. 1	30.69%	#4
Apache Config. No. 2	49.35%	#1
Apache Config. No. 3	40.24%	#2
Apache Config. No. 4	35.24%	#3
Apache Config. No. 5	27.90%	#5
Apache Config. No. 6	27.50%	#6
Apache Config. No. 7	11.66%	#9
Apache Config. No. 8	18.05%	#8
Apache Config. No. 9	8.48%	#10
Apache Config. No. 10	23.33%	#7

It is important to note that AHP assumes no interdependence between criteria, meaning that each feature is evaluated independently of others, which is crucial to the accuracy of the final results.

### TOPSIS application methodology

The TOPSIS application for prioritising configurations in feature models consists of the steps described in “TOPSIS”.

For the Apache Tomcat server example, Phase 2 starts with the collection of the expert’s preferences, which are expressed as qualitative criteria. These criteria are then transformed into quantitative criteria so that TOPSIS can use them to subsequently calculate the distances of the alternatives to the positive and negative ideal solution (PIS and NIS, respectively).

In this transformation, the numerical scales are derived directly from the expert’s ranking of the instances. The expert explicitly establishes an order of preference for the instances of each criterion, and these rankings are then mapped onto ordinal scales where the least preferred option is assigned the lowest value (*e.g*., 1) and the most preferred option the highest. This approach ensures that the numerical values correspond faithfully with the expert’s qualitative judgment, with no additional weighting or transformation beyond the direct ranking.

This transformation is shown in Table 8. If any of the selected alternatives does not have a value assigned for one or more of the identified criteria, a value of 0 is set.

**Table 8:** Numeric scales assigned to criteria.

**(a) ClientAuth instances**	
False	1
Want	2
True	3
**(b) KeyStore and Trust instances**	
PKCS11	1
JKS	2
PKCS12	3
**(c) Protocol version instances**	
v1	1
v1.1	2
v1.2	3
v1.3	4
**(d) Protocol numeric scale with multiple instances**	
v1	1
v1.1	2
v1.2	3
v1.3	4
v1.2 & v1.3	3.5

Since the expert’s preference rankings are given for individual *Protocol* versions, and the OR-feature type was set to “average,” the value for *v1.2 & v1.3* was automatically computed as the arithmetic mean of the values assigned to *v1.2* and *v1.3*, with Table 8c resulting in Table 8d. This ensures consistency in cases where multiple instances are valid within a configuration. Afterward, in Phase 3, the weights of the criteria need to be determined, assigning the weights as shown in Table 9.

**Table 9:** Assignment of weights to the criteria.

ClientAuth	35%
KeyStore	15%
Trust	30%
Protocol	20%

Based on this information, both the PIS and the NIS and their distances to each alternative are calculated, and these distances are used to obtain the ranking in Table 10. *R* represents the relative closeness or similarity index of an alternative to the ideal solution. The closer *R* is to 1, the closer the evaluated alternative is to the positive ideal solution (PIS). The closer *R* is to 0, the closer the alternative is to the negative ideal solution (NIS).

**Table 10:** Ranking of apache feature model alternatives obtained with TOPSIS.

Alternatives	R	Ranking
Apache Config. No. 1	0.5303	#5
Apache Config. No. 2	0.7972	#1
Apache Config. No. 3	0.7452	#2
Apache Config. No. 4	0.6684	#3
Apache Config. No. 5	0.5791	#4
Apache Config. No. 6	0.4596	#6
Apache Config. No. 7	0.1546	#9
Apache Config. No. 8	0.2712	#8
Apache Config. No. 9	0.0000	#10
Apache Config. No. 10	0.3629	#7

### VIKOR application methodology

The VIKOR application for prioritising configurations in feature models is performed as described in “VIKOR”. In Phase 2, the process begins with the introduction of expert preferences, expressed as qualitative criteria. For the Apache Tomcat server example, the numerical scales assigned in the TOPSIS application methodology are reused. The best and worst values for each criterion are also determined at this stage.

In Phase 3, the VIKOR method calculates the utility and regret measures for each alternative and produces the ranking of the configurations. The weighting of the criteria is the same as those established in the TOPSIS application methodology. Applying the method to the example of the Apache Tomcat server gives the ranking of the alternatives shown in Table 11 is obtained. *Q* is a key value used to rank alternatives and determine the compromise solution, *i.e*., a lower value indicates a better alternative.

**Table 11:** Ranking of Apache feature model alternatives obtained with VIKOR.

Alternatives	*Q*	Ranking
Apache Config. No. 1	0.3841	#5
Apache Config. No. 2	0.0200	#1
Apache Config. No. 3	0.0529	#2
Apache Config. No. 4	0.3212	#3
Apache Config. No. 5	0.3841	#6
Apache Config. No. 6	0.3311	#4
Apache Config. No. 7	0.8147	#9
Apache Config. No. 8	0.6294	#8
Apache Config. No. 9	1,000	#10
Apache Config. No. 10	0.4241	#7

### Implementation and package for replicability

This section provides a comprehensive overview of the implementation and practical details of the *decision support module* within the AMADEUS framework (Varela-Vaca et al., 2020). This includes the rationale behind its development, validation aspects, and key implementation details to facilitate replication and application by other researchers and practitioners.

#### Rationale for the framework

The AMADEUS framework provides experts with tools to identify and evaluate multiple system configurations. However, it lacks an automated prioritisation system to determine the optimal order in which configurations should be addressed. Without a structured decision-making approach, prioritisation is often based on basic criteria, overlooking key factors such as impact, risk, complexity, or cost. These factors are essential for optimising resources and ensuring that critical configurations receive priority.

To address this gap, we have developed a *decision support module* within AMADEUS, enabling automated and systematic configuration prioritisation using multi-criteria decision-making (MCDM) methods. This enhancement extends the framework’s capabilities, providing a structured approach to ranking configurations based on multiple user-defined criteria.

The integration of MCDM methods offers several advantages:

(1) Comprehensive evaluation. MCDM allows multiple factors to be analysed simultaneously, a crucial aspect in security and IT management where decisions must balance impact, risk, and mitigation costs.
(2) Optimised resource allocation. Experts can prioritise the configurations that pose the greatest risk or provide the highest benefit, ensuring an efficient use of time and effort.
(3) Scalability and adaptability. The framework remains effective even as the number of configurations grows, supporting structured assessments that adapt to evolving IT infrastructures.

By incorporating MCDM, AMADEUS enhances decision-making for product variability management, allowing experts to adjust prioritisation criteria as needed. This improvement not only enables more efficient configuration management but also ensures a proactive, adaptive response to organisational requirements.

#### Discussion on the validity and limitations of the framework

The validity of this framework lies in its ability to enhance configuration prioritisation through the integration of MCDM methods and a structured decision-support process. The *decision support module*, incorporated into AMADEUS, takes advantage of well-established MCDM techniques—AHP, TOPSIS, and VIKOR—which have been extensively validated in various decision-making domains. Their inclusion ensures a systematic and reliable prioritisation approach.

To assess its practical applicability, the framework has been tested using real-world feature models, such as the SSL/TLS configuration model (cf. Results). These case studies confirm that the module effectively processes user-defined criteria and preferences, producing coherent and interpretable rankings. Furthermore, AMADEUS ensures consistency and completeness by resolving ranking discrepancies, allowing for the prioritisation of configurations under identical conditions and evaluation criteria. Its adaptability ensures that all relevant factors can be considered, reducing the risk of overlooking key decision elements.

Despite its advantages, the framework has some inherent limitations. The effectiveness of MCDM methods depends on the correct definition of criteria and weighting, which introduces a degree of subjectivity. Since different experts may assign different importance levels to criteria, results can vary across implementations. Improperly defined priorities could lead to suboptimal configurations that do not align with organisational needs.

Additionally, the initial setup—defining MCDM criteria and customising feature models—requires a certain level of expertise. Organisations lacking knowledgeable personnel may struggle to properly configure the tool, potentially limiting adoption and effectiveness.

In large-scale environments, managing a high number of configurations can pose challenges in computational efficiency. However, while the number of valid configurations grows exponentially as features increase, the computational cost of our approach is primarily influenced by the number of selected criteria and their instances, rather than the total number of configurations. Since the core processing relies on pairwise comparisons, the complexity is driven by expert-defined preferences rather than FM size. Nevertheless, in cases involving extremely large FMs, modularisation techniques can be employed to divide the FM into smaller, more manageable sub-models, improving efficiency and maintainability.

#### Discussion on implementation

The *decision support module* has been implemented as part of the AMADEUS framework available at https://doi.org/10.5281/zenodo.14870223. The implementation includes clear installation instructions, usage guidelines and a list of prerequisites detailed in the README.md file, ensuring that users can set up the module with minimal effort. In addition, a comprehensive usage example is provided in the DECISION_TREES.md file, which guides users through the process of applying MCDM methods to a feature model and obtaining configuration rankings. This documentation, together with the automated processes embedded in the module, ensures that the application of AHP, TOPSIS and VIKOR is straightforward and accessible. The module supports a range of input formats and provides clear output that highlights the prioritisation of configurations based on user-defined criteria and preferences.

Additionally, two detailed markdown files have been included in the repository to guide users through the application of the MCDM methods on the Apache Tomcat server and the TLS/SSL examples (cf. Results). This files contain step-by-step instructions, input data, and matrices to facilitate replicability. The files are available at https://doi.org/10.5281/zenodo.14870223.

Originally, AMADEUS includes validation mechanisms to ensure that each generated FM is consistent and represents only valid configurations. This includes testing valid products and invalidating spurious configurations. Now, AMADEUS includes the *decision support module*, which provides reasoning operations to identify priority configurations according to the defined criteria. To minimise computation time in systems with dynamic configurations, incremental reasoning has been introduced to update only those parts of the FM affected by recent changes, avoiding the need to rebuild the entire model.

## Informed decisions: in-depth examination of ranking discrepancies

When there are discrepancies between the rankings generated by different MCDM techniques, it becomes essential to provide the user with a systematic approach to selecting the most appropriate configuration. In this phase, our goal is to support informed decision-making by analysing configurations that occupy similar or identical positions in the rankings, identifying key differences and similarities between them, and presenting this information in a manner that assists the user in making a choice.

It is important to acknowledge that the attainment of disparate rankings may be attributable to the weighting assigned to the criteria in the various techniques (obtained in AHP from the comparisons and established for TOPSIS and VIKOR), which may differ and result in analogous yet non-identical solutions. For the Apache example, the weights obtained by AHP for *ClientAuth*, *Trust*, *Protocol* and *KeyStore* are 37.01%, 34.51%, 18.50% and 9.99% respectively. For VIKOR and TOPSIS the established weights are 35%, 30%, 20%, and 15%. Despite the utilisation of identical weights by VIKOR and TOPSIS, the attainment of disparate rankings remains a possibility, as articulated in the work by Shekhovtsov & Salabun (2020).

The process of analysing discrepancies is divided into three key steps:

1. **Identifying configurations with ranking discrepancies**

The initial step is to identify configurations that are similarly ranked by different MCDM methods, yet differ in specific criteria or instances. For example, Table 12 shows configurations ranked by AHP, TOPSIS, and VIKOR. In this example, the configurations ranked #4, #5, and #6 show notable discrepancies, making them candidates for deeper analysis.

**Table 12:** Ranking of Apache feature model.

Ranking	Apache Config. No.		
	AHP	TOPSIS	VIKOR
#1	2	2	2
#2	3	3	3
#3	4	4	4
#4	1	5	6
#5	5	1	1
#6	6	6	5
#7	10	10	10
#8	8	8	8
#9	7	7	7
#10	9	9	8

2. **Analysing criteria and instances**

We then focus on the specific criteria that influence the discrepancies in these rankings. By examining the feature model and analysing the instances of criteria within each configuration, patterns or trade-offs can be identified. For example, while Apache Config. No. 1 may prioritise certain features (*e.g*., *ClientAuth*), Apache Config. No. 5 and 6 may prioritise others (*e.g*., *Trust* or *Protocol*). The disparities in feature prioritisation and their repercussions on the aggregate ranking are emphasised to assist the user in evaluating their options based on their particular preferences.

**Figure 5:**
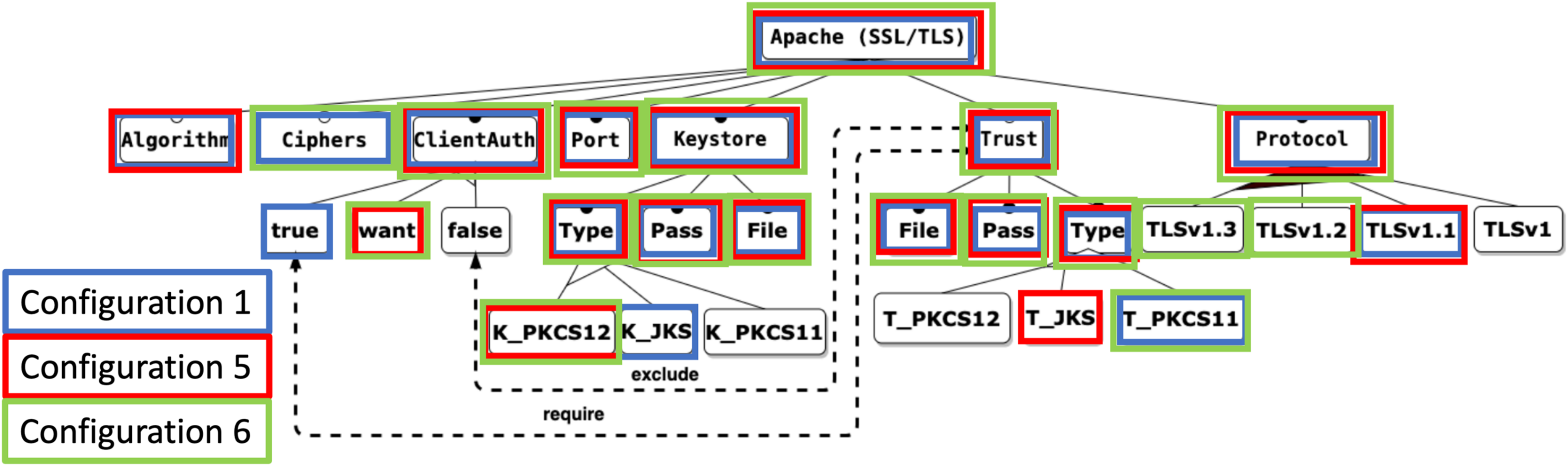
Apache feature model coloured with configurations.

However, as shown in Fig. 5, the visual representation of features in configurations does not invariably facilitate straightforward decision-making, as mandatory features and specific feature constraints (*e.g*., XOR relations) can impede interpretation.

3. **Generating an informed decision-making support table.**

To provide a more systematic and comprehensible approach, we propose to represent the criteria and instances in a structured decision support table. This table organises the configurations based on the values assigned to each criterion and the deviations from optimal values (according to the user-defined preferences) as presented in Table 13. These deviations are calculated as percentages based on the numerical scales predefined by the expert for each criterion (within the preferences *P* in the global process). For example, the expert may assign a numerical value of 2 to *true*, 1 to *want*, and 0 to *false* for the criterion *ClientAuth*. These values reflect the expert’s priorities, and the percentage deviation indicates how far each configuration is from the optimal value (the one with the highest assigned number).

**Table 13:** Informed decision-making support table showing percentage deviations for each configuration.

Apache Config. No.	ClientAuth	KeyStore	Trust	Protocol	Overall deviation
1	True (0%)	JKS (50%)	PKCS11 (100%)	1.1 (66%)	54%
5	Want (50%)	PKCS12 (0%)	JKS (50%)	1.1 (66%)	41.5%
6	Want (50%)	PKCS12 (0%)	PKCS11 (100%)	1.2 & 1.3 (16%)	41.5%

Each configuration is assigned a score based on a percentage scale and the *overall deviation* is calculated as the average of the individual deviations across all criteria. This overall metric provides a clear representation of how close or far each configuration is from meeting the expert’s objectives. A more detailed discussion of the robustness and justification of the overall deviation metric is provided in “Robustness and Justification of the Overall Deviation Metric”.

As demonstrated in the table, Apache Config. No. 1 optimises *ClientAuth*, yet assigns a lower priority to *Trust* and *Protocol*. In contrast, Apache Config. No. 5 and 6 offer a balanced trade-off. The percentage deviation for each criterion reflects the configuration’s deviation from the expert’s preferred option. For example, in the case of *Protocol*, Apache Config. No. 1 deviates by 66% (using version 1.1), whilst Apache Config. No. 6 deviates by a much smaller percentage of 16% (with versions 1.2 & 1.3).

In terms of *Overall deviation*, Apache Config. No. 1 has 54%, indicating a greater misalignment with the expert’s priorities compared to Apache Config. No. 5 and 6, which have lower overall deviations of 41.5%. However, the specific criteria deviations reveal additional trade-offs. Specifically, Apache Config. No. 1 excels in *ClientAuth* but performs poorly in *Trust* and *Protocol*. In contrast, Apache Config. No. 5 and 6 exhibit more balanced deviations across the criteria.

By presenting the information in this manner, the expert is able to effortlessly compare configurations and balance the trade-offs according to the specific criteria that are most significant in the given context.

In summary, this section provides a systematic approach to addressing discrepancies in rankings generated by different MCDM methods. The process is broken down into three steps, ensuring that the expert has a clear and actionable understanding of the trade-offs involved. The incorporation of percentage deviations and the overall deviation metric provides a quantitative framework for the comparison of configurations, thereby highlighting their alignment with the expert’s preferences. This structured approach facilitates a more informed and confident decision-making process, even in scenarios involving conflicting rankings.

### Robustness and justification of the overall deviation metric

The overall deviation metric is a robust tool for quantifying how closely each configuration aligns with the expert’s preferences. While the individual deviations offer insight into specific criteria, the overall deviation aggregates this information to provide a comprehensive view of each configuration’s suitability.

**Formal Justification:**

(1) Interpretability: By expressing deviations as percentages, the overall deviation is rendered comprehensible and intuitively graspable in terms of how far a configuration deviates from optimal preferences. This accessibility extends beyond technical experts to decision-makers who may not possess extensive mathematical proficiency.
(2) Criterion weight integration: The metric inherently incorporates the relative importance of each criterion. The relative importance of each criterion is determined by the expert, with higher-priority criteria contributing more significantly to the overall deviation. This ensures that configurations misaligned on crucial criteria are penalised appropriately.
(3) Consistency across methods: Since the preferences are consistent across AHP, TOPSIS, and VIKOR, the overall deviation provides a unified framework to compare results from these different methods. This enables a coherent evaluation of discrepancies and facilitates the identification of the most suitable configurations.
(4) Adaptability to feature models: Feature models often include both qualitative and quantitative attributes. The overall deviation is capable of handling both types of attributes by translating qualitative preferences into numerical scales and combining them in a way that respects the model’s structure (*e.g*., handling OR relationships through weighted averages or other aggregation functions).

**Comparison with alternative metrics: **While alternative distance-based metrics, such as Euclidean distance or Manhattan distance, could be considered, they may not offer the same level of interpretability or alignment with expert-defined preferences. These traditional distance metrics treat all deviations equally, without considering the relative importance of criteria. In contrast, the overall deviation is tailored to the decision-making context by emphasizing expert-driven priorities.

**Empirical validation: **The robustness of the overall deviation is demonstrated through the Apache Tomcat server and SSL/TLS examples in “Informed Decisions: In-Depth Examination of Ranking Discrepancies” and “Results”. In both cases, the configurations with lower overall deviations correspond to those ranked higher by multiple MCDM methods, validating the metric’s effectiveness in highlighting the most aligned configurations. Furthermore, the metric’s ability to clearly differentiate configurations with subtle differences supports its practical utility in complex decision-making scenarios.

## Results

**Figure 6:**
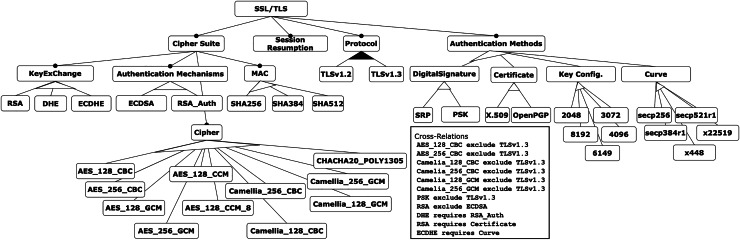
SSL/TLS feature model.

To demonstrate the application and evaluation of the decision-making techniques presented in this work, we have applied them to a more complex example. Specifically, the SSL/TLS feature model is used, which is related to the field of cybersecurity and is based on the contribution of Varela-Vaca et al. (2019). This model represents the attributes required to configure certificates, keystores, ciphers, and key sizes for different versions of the SSL/TLS protocol, as shown in Fig. 6. The model consists of 48 features, including eight mandatory relations, two or-alternative relations, eight alternative relations, and four require and eight exclude constraints.

The evaluation process follows the four phases described in the proposal: (1) inputs and model generation, (2) preferences setting, (3) application of MCDM methods, and (4) resolution of rankings discrepancies. The application of these phases to the SSL/TLS feature model is detailed below.

### Phase 1. Inputs and model generation

For the SSL/TLS Feature Model example, the input parameters are as follows:

• Feature model: as shown in Fig. 6.
• Criteria and instances: of the 48 features in the model, nine are identified as criteria, each with associated instances representing possible values:
  – Algorithm for Key Generation (KeyExChange): RSA, DHE, or ECDHE.
  – Authentication method to use (AuthenticationMechanisms): ECDSA or RSA_Auth.
  – Encryption algorithm (Cipher): AES_128_CBC, AES_256_CBC, AES_128_GCM, AES_256_GCM, AES_128_CCM, AES_128_CCM_8, Camellia_128_CBC, Camellia_256_CBC, Camellia_128_GCM, Camellia_256_GCM, or CHACHA20_POLY1305.
  – Algorithm for message encryption and integrity provision (Mac): SHA256, SHA384, or SHA512.
  – SSL/TLS protocol version (Protocol): 1.2 or 1.3.
  – Type of digital signature support instead of certificates (DigitalSignature): SRP or PSK.
  – Type of supported certificate (Certificate): X.509 or OpenPGP.
  – Key configuration (KeyConfig): 2048, 3072, 4096, 6149, or 8192.
  – Function for generating elliptic curve keys (Curve): secp256, secp384r1, x448, x22519, or secp521r1.

• Valid configurations: Using CyberSPL (Varela-Vaca et al., 2019), 1,482 configurations were generated, from which only 10 configurations were selected as alternatives to be evaluated, shown in Table 14.

**Table 14:** Selected configurations to prioritise.

Config. No.	SSL/TLS product configuration
1	SSL_TLS–Cipher–KeyExChange–RSA–AuthenticationMechanisms–RSA_Auth–Cipher–AES_128_CBC–MAC–SHA384–SessionResumption–Protocol–TLSv1_2–AuthenticationMethods–Certificate–X_509
2	SSL_TLS–Cipher–KeyExChange–DHE–AuthenticationMechanisms–RSA_Auth–Cipher–Camellia_256_GCM–MAC–SHA512–SessionResumption–Protocol–TLSv1_2–AuthenticationMethods–DigitalSignature–PSK
3	SSL_TLS–Cipher–KeyExChange–ECDHE–AuthenticationMechanisms–ECDSA–Cipher–AES_128_GCM–MAC–SHA256–SessionResumption–Protocol–TLSv1_3–AuthenticationMethods–Curve–secp256
4	SSL_TLS–Cipher–KeyExChange–RSA–AuthenticationMechanisms–RSA_Auth–Cipher–Camellia_256_CBC–MAC–SHA512–SessionResumption–Protocol–TLSv1_2–AuthenticationMethods–Certificate–OpenPGP
5	SSL_TLS–Cipher–KeyExChange–DHE–AuthenticationMechanisms–RSA_Auth–Cipher–AES_128_CCM–MAC–SHA384–SessionResumption–Protocol–TLSv1_3–AuthenticationMethods–KeyConfig–KC_6149
6	SSL_TLS–Cipher–KeyExChange–ECDHE–AuthenticationMechanisms–ECDSA–Cipher–AES_128_CCM_8–MAC–SHA512–SessionResumption–Protocol–TLSv1_3–AuthenticationMethods–Curve–secp384r1
7	SSL_TLS–Cipher–KeyExChange–RSA–AuthenticationMechanisms–RSA_Auth–Cipher–Camellia_128_CBC–MAC–SHA512–SessionResumption–Protocol–TLSv1_2–AuthenticationMethods–Certificate–X_509
8	SSL_TLS–Cipher–KeyExChange–DHE–AuthenticationMechanisms–RSA_Auth–Cipher–AES_256_GCM–MAC–SHA256–SessionResumption–Protocol–TLSv1_3–AuthenticationMethods–KeyConfig–KC_3072
9	SSL_TLS–Cipher–KeyExChange–ECDHE–AuthenticationMechanisms–ECDSA–Cipher–Camellia_128_GCM–MAC–SHA384–SessionResumption–Protocol–TLSv1_2–AuthenticationMethods–Curve–secp521r1
10	SSL_TLS–Cipher–KeyExChange–RSA–AuthenticationMechanisms–RSA_Auth–Cipher–CHACHA20_POLY1305–MAC–SHA256–SessionResumption–Protocol–TLSv1_3–AuthenticationMethods–Certificate–OpenPGP

### Phase 2. Preferences setting

In this phase, preferences were defined for each criterion and its corresponding instances. These preferences were determined by pairwise comparisons (AHP), and by assigning weights to each criterion and instances in the application of TOPSIS and VIKOR.

• AHP: The pairwise comparison matrices are shown in Table 15. Weights are assigned to criteria and instances based on these pairwise comparison matrices, with the following weights for each criterion:
  – KeyExChange (26.53%)
  – Authentication Mechanism (10.97%)
  – Cipher (14.82%)
  – Mac (6.95%)
  – Other criteria (Protocol, DigitalSignature, Certificate, KeyConfig, Curve) equally weighted at 8.15% each.

**Table 15:** Pairwise comparison matrices.

(a) Criteria									
	KeyExChange	Authentication mechanisms	Cipher	Mac	Protocol	Digital signature	Certificate	KeyConfig	Curve
KeyExChange	1	3	2	5	3	3	3	3	3
AuthenticationMechanisms	1/3	1	2	2	1	1	1	1	1
Cipher	1/2	1/2	1	3	2	2	2	2	2
Mac	1/5	1/2	1/3	1	1	1	1	1	1
Protocol	1/3	1	1/2	1	1	1	1	1	1
DigitalSignature	1/3	1	1/2	1	1	1	1	1	1
Certificate	1/3	1	1/2	1	1	1	1	1	1
KeyConfig	1/3	1	1/2	1	1	1	1	1	1
Curve	1/3	1	1/2	1	1	1	1	1	1

(b) KeyExChange instances			
	RSA	DHE	ECDHE
RSA	1	2	1/2
DHE	1/2	1	1/4
ECDHE	2	4	1

(c) AuthenticationMechanism instances		
	ECDSA	RSA_Auth
ECDSA	1	2
RSA_Auth	1/2	1

(d) Cipher instances											
	AES_128_CBC	AES_256_CBC	AES_128_GCM	AES_256_GCM	AES_128_CCM	AES_128_CCM_8	Camellia_128_CBC	Camellia_256_CBC	Camellia_128_GCM	Camellia_256_GCM	CHACHA20_POLY1305
AES_128_CBC	1	1/2	1/3	1/4	1/5	1/6	1/7	1/8	1/9	1/10	1/11
AES_256_CBC	2	1	1/2	1/3	1/4	1/5	1/6	1/7	1/8	1/9	1/10
AES_128_GCM	3	2	1	1/2	1/3	1/4	1/5	1/6	1/7	1/8	1/9
AES_256_GCM	4	3	2	1	1/2	1/3	1/4	1/5	1/6	1/7	1/8
AES_128_CCM	5	4	3	2	1	1/2	1/3	1/4	1/5	1/6	1/7
AES_128_CCM_8	6	5	4	3	2	1	1/2	1/3	1/4	1/5	1/6
Camellia_128_CBC	7	6	5	4	3	2	1	1/2	1/3	1/4	1/5
Camellia_256_CBC	8	7	6	5	4	3	2	1	1/2	1/3	1/4
Camellia_128_GCM	9	8	7	6	5	4	3	2	1	1/2	1/3
Camellia_256_GCM	10	9	8	7	6	5	4	3	2	1	1/2
CHACHA20_POLY1305	11	10	9	8	7	6	5	4	3	2	1

(e) MAC instances			
	SHA256	SHA384	SHA512
SHA256	1	1/2	1/4
SHA384	2	1	1/2
SHA512	4	2	1

(f) Protocol instances		
	v1.2	v1.3
v1.2	1	1/2
v1.3	2	1

(g) DigitalSignature instances		
	SRP	PSK
SRP	1	1/2
PSK	2	1

(h) KeyConfig instances					
	Key size 2048	Key size 3072	Key size 4096	Key size 6149	Key size 8192
Key size 2048	1	1/2	1/3	1/4	1/5
Key size 3072	2	1	1/2	1/3	1/4
Key size 4096	3	2	1	1/2	1/3
Key size 6149	4	3	2	1	1/2
Key size 8192	5	4	3	2	1

(i) Certificate instances		
	X.509	OpenPGP
X.509	1	2
OpenPGP	1/2	1

(j) Curve instances					
	secp256	secp384r1	x448	x22519	secp521r1
secp256	1	1/2	2	4	1/4
secp384r1	2	1	4	6	1/2
x448	1/2	1/4	1	2	6
x22519	1/4	1/6	1/2	1	1/8
secp521r1	4	2	1/6	8	1

• TOPSIS and VIKOR: the qualitative criteria are transformed into quantitative criteria by assigning numerical scales, as shown in Table 16. Weights of the criteria are set as follows:
  – KeyExChange (20%)
  – Authentication Mechanism (10%)
  – Cipher (15%)
  – Mac (5%)
  – Other criteria were equally weighted at 10% each.

**Table 16:** Numeric scales assigned to criteria.

**(a) KeyExChange criterion**	
DHE	1
RSA	2
ECDHE	3
**(b) AuthenticationMechanism criterion**	
RSA_Auth	1
ECDSA	2
**(c) Mac criterion**	
SHA256	1
SHA384	2
SHA512	3
**(d) Protocol criterion**	
1.2	1
1.3	2
**(e) DigitalSignature criterion**	
SRP	1
PSK	2
**(f) Certificate criterion**	
OpenPGP	1
X.509	2
**(g) Cipher criterion**	
AES_128_CBC	1
AES_128_GCM	2
AES_128_CCM	3
Camellia_128_CBC	4
Camellia_128_GCM	5
AES_128_CCM_8	6
AES_256_CBC	7
Camellia_256_CBC	8
AES_256_GCM	9
Camellia_256_GCM	10
CHACHA20_POLY1305	11
**(h) KeyConfig criterion**	
2048	1
3072	2
4096	3
6149	4
8192	5
**(I) Curve criterion**	
x22519	1
x448	2
secp256	3
secp384r1	4
secp521r1	5

It is important to note that the number of selected alternatives (*i.e*., valid configurations) does not significantly affect the computational cost in the initial processing stages. The complexity of the proposed approach primarily depends on the number of criteria and their possible values, rather than the number of alternatives. Specifically, the pairwise comparison process for criteria and instances in AHP involves filling a comparison matrix of size $n \times n$, leading to a complexity of $O({n^{2}})$. However, the full weight computation in AHP, including eigenvalue estimation, is more computationally demanding and is further discussed in “Computational complexity and discrepancy resolution efficiency”. In contrast, TOPSIS and VIKOR require numerical scales instead of pairwise comparisons, and their complexity is approximately $O(mn)$, where $m$ is the number of configurations and $n$ is the number of criteria. Since n is typically much smaller than $m$, our method remains feasible even for large feature models. This ensures a consistent and efficient application of AHP, TOPSIS, and VIKOR, focusing on the hierarchical relationships between attributes and maintaining robustness across different problem sizes.

### Phase 3. Application of the MCDM methods

In this phase, the three MCDM methods—AHP, TOPSIS, and VIKOR—were applied, resulting in three different rankings of the 10 configurations. The AHP method calculates the priority of each alternative based on the weights and instances of the criteria, producing the ranking shown in Table 17. TOPSIS was applied using the given weights, producing the ranking shown in Table 18. The VIKOR method has been applied, giving the rank shown in Table 19.

**Table 17:** Ranking of the SSL/TLS feature model alternatives AHP.

Alternatives	Priority	Ranking
SSL/TLS Config. No. 1	21.55%	8
SSL/TLS Config. No. 2	22.26%	6
SSL/TLS Config. No. 3	30.95%	2
SSL/TLS Config. No. 4	21.79%	7
SSL/TLS Config. No. 5	17.32%	9
SSL/TLS Config. No. 6	35.00%	1
SSL/TLS Config. No. 7	23.81%	5
SSL/TLS Config. No. 8	16.86%	10
SSL/TLS Config. No. 9	30.36%	3
SSL/TLS Config. No. 10	24.45%	4

**Table 18:** Ranking of SSL/TLS feature model alternatives TOPSIS.

Alternatives	R	Ranking
SSL/TLS Config. No. 1	0.3344	9
SSL/TLS Config. No. 2	0.4128	5
SSL/TLS Config. No. 3	0.4272	4
SSL/TLS Config. No. 4	0.3643	7
SSL/TLS Config. No. 5	0.3175	10
SSL/TLS Config. No. 6	0.4996	1
SSL/TLS Config. No. 7	0.3733	6
SSL/TLS Config. No. 8	0.3574	8
SSL/TLS Config. No. 9	0.4796	2
SSL/TLS Config. No. 10	0.4540	3

**Table 19:** Ranking of SSL/TLS feature model alternatives VIKOR.

Alternatives	*Q*	Ranking
SSL/TLS Config. No. 1	0.9500	10
SSL/TLS Config. No. 2	0.8579	7
SSL/TLS Config. No. 3	0.3429	3
SSL/TLS Config. No. 4	0.7460	6
SSL/TLS Config. No. 5	0.9289	9
SSL/TLS Config. No. 6	0.0000	1
SSL/TLS Config. No. 7	0.7392	5
SSL/TLS Config. No. 8	0.8934	8
SSL/TLS Config. No. 9	0.2842	2
SSL/TLS Config. No. 10	0.4855	4

### Phase 4. Resolving discrepancies in rankings

For the SSL/TLS example, the method calculates the deviation for configurations that appear in different positions in the rankings. The informed decision-making support table summarises how far each configuration deviates from the optimal values set by the respective methods, intending to provide more information to the user when deciding between configurations.

In the case presented in Table 20, the first configuration selected is the same for all three techniques SSL/TLS Config. No. 6. However, the following positions in the three rankings do not correspond to SSL/TLS Config. No. 3, 9 and 10 appear in a different order. Following the method explained in “Informed Decisions: In-Depth Examination of Ranking Discrepancies”, we extract the main differences between these configurations by analysing their respective criteria values and calculating the overall deviation from ideal values. For this phase, we consider the exclusive OR relationship between the criteria ‘DigitalSignature’, ‘Certificate’, ‘KeyConfig’, and ‘Curve’. This means that only one of them can be instantiated and that they should be treated as one single criterion when calculating the overall deviation.

**Table 20:** Ranking of SSL/TLS alternatives.

SSL/TLS configurations		
AHP	TOPSIS	VIKOR
6	6	6
3	9	9
9	10	3
10	3	10
7	2	7
2	7	4
4	4	2
1	8	8
5	1	5
8	5	1

Table 21 summarises the results, where each row corresponds to a configuration and each column represents a criterion, with the last column showing the overall deviation. The percentages shown next to each instance represent the deviation of that instance from the optimal value for its criterion, while the overall deviation reflects how far the entire configuration is from the ideal configuration based on the expert-defined preferences.

**Table 21:** Informed decision-making support table for SSL/TLS Config. No. 3, 9, and 10.

SSL/TLS Config. No.	KeyExChange	Cipher	AuthMech	Protocol	DigitalSign/Cert/KeyConf/Curve	Mac	Overall Deviation
3	ECDHE (0%)	AES 128 GCM (90%)	EDCSA (0%)	TLSv1.3 (0%)	secp256 (50%)	SHA256 (100%)	44.17%
9	ECDHE (0%)	Camellia 128 GCM (60%)	ECDSA (0%)	TLSv1.2 (100%)	secp521r1 (0%)	SHA384 (50%)	35.00%
10	RSA (50%)	CHACHA20_POLY1305 (0%)	RSA Auth (100%)	TLSv1.3 (0%)	OpenPGP (100%)	SHA256 (100%)	58.33%

As shown in Table 21, SSL/TLS Config. No. 9 has the lowest overall deviation, which is consistent with its higher ranking in some of the techniques used. SSL/TLS Config. No. 3 follows with a moderate deviation and SSL/TLS Config. No. 10 has the highest variance due to its lower performance in certain key criteria.

### Computational complexity and discrepancy resolution efficiency

This section provides an analysis of the computational complexity of the proposed framework and discusses the efficiency of resolving discrepancies when different MCDM methods produce divergent rankings.

**Computational complexity of MCDM methods. **The computational complexity of the framework varies across its phases. In Phase 2 (Preferences Setting), the main cost comes from the number of criteria and their possible values, as pairwise comparisons and numerical scales are defined independently of the number of configurations. However, in Phase 3 (Application of MCDM methods), the number of configurations affects execution time in TOPSIS and VIKOR, as both methods evaluate all alternatives against the defined criteria. This is reflected in Table 22, which summarizes the expected growth trends in execution time based on the theoretical complexity of AHP, TOPSIS, and VIKOR.

**Table 22:** Computational complexity of MCDM methods.

Scenario	Criteria (n)	Configurations (m)	Expected execution time trend
Small	Low (<10)	Few (<50)	Fast: execution in milliseconds for all methods
Medium	Moderate (10–20)	Moderate (50–200)	Manageable: AHP grows noticeably, but all remain feasible
Large	High (20–30)	High (200–500)	Performance impact: AHP requires optimization, TOPSIS/VIKOR remain scalable
Very large	Very high (>30)	Very high (>500)	Challenging: AHP may become impractical, alternative strategies needed

The full execution of AHP involves both pairwise comparisons ( $O({n^{2}})$) and eigenvalue computation to derive priorities ( $O({n^{3}})$). The latter step is the most computationally expensive, making the overall complexity of AHP approximately $O({n^{3}})$. In contrast, TOPSIS and VIKOR require computing distances to ideal solutions, resulting in a complexity of $O(mn)$, which scales linearly with the number of configurations. The main computational effort in TOPSIS and VIKOR lies in calculating the weighted normalised decision matrix and determining the positive and negative ideal solutions.

**Efficiency of discrepancy resolution in Phase 4. **The computational effort in Phase 4 is primarily dictated by the construction of the overall deviation table (*e.g*., Table 21). This process involves the calculation of percentage deviations for each configuration in relation to the ideal values defined by the expert. Given that this process entails elementary arithmetic operations over a predefined set of criteria and configurations, its complexity is linear with respect to n and m (*i.e*., $O(nm)$), ensuring computational efficiency.

Additionally, the approach ensures that the resolution process remains scalable by using structured numeric preference scales (as detailed in “TOPSIS application methodology”). This approach facilitates the rapid identification of the most significant differences between configurations, thereby obviating the need for exhaustive manual comparison by the expert.

In summary, our framework maintains computational feasibility across all phases, ensuring that even with large feature models, the prioritisation and discrepancy resolution steps remain practical for real-world applications.

## Discussion

To the best of our knowledge, this is the first approach that applies multiple MCDM techniques to prioritize configurations derived from feature models. The existing tools focus on either feature selection or configuration analysis, but do not offer systematic prioritisation or reconciliation of discrepancies between different decision-making methods. This positions the present framework as a novel contribution to the field of feature model-based decision support.

Beyond its novelty, the framework has practical implications for industries and domains that rely on complex system configurations. By enabling systematic prioritisation, the approach ensures that configurations meet user-defined criteria, ultimately improving product quality and development efficiency. This has direct implications in areas such as cloud computing, IoT systems, embedded software design and more, where feature-rich products require careful selection of optimal configurations.

The results obtained from the application of the proposed MCDM-based configuration prioritisation methodology highlight several important findings and considerations. The methodology is effective in generating comprehensive rankings using AHP, TOPSIS and VIKOR, each of which brought unique perspectives to the prioritisation process.

The MCDM methods employed in this study offer distinct advantages, depending on the decision-making context. AHP is particularly beneficial when hierarchical structuring of criteria and pairwise comparisons are required, making it well-suited for cases where experts can systematically evaluate preferences. However, AHP may be less practical when the number of criteria or alternatives is very large, due to the increasing complexity of the pairwise comparisons. In contrast, TOPSIS is advantageous in scenarios where a clear distinction between the best and worst alternatives is essential, as it directly evaluates each alternative’s closeness to the ideal solution. However, its reliance on normalisation techniques means that its results can be influenced by the range of values assigned to criteria. In contrast, VIKOR is particularly useful when trade-offs between multiple criteria need to be explicitly considered, as it introduces a compromise ranking based on regret and satisfaction measures, making it suitable for scenarios where a balance between the best and worst cases is required. Given these differences, our approach benefits from combining these methods.

An important implication of this study is that applying different MCDM methods to the same problem can yield different results due to differences in how each technique weights and processes the criteria. This highlights the need for informed decision support tools, such as those proposed in Phase 4, to help experts understand and resolve these differences.

A key strength of this approach is its adaptability to different decision scenarios, including complex systems with intricate feature models. By systematically structuring criteria and alternatives, experts are provided with a clear path to prioritise configurations according to defined preferences. The integration of automated tools within the AMADEUS framework further enhances this process by simplifying implementation and ensuring reproducibility.

Despite these strengths, there are limitations. The reliance on expert input to define the criteria weights and interpret the results introduces a subjective element that can affect the consistency of the rankings. Similarly, although the methodology is scalable, the main complexity lies in the amount of input required from the expert. As more features are selected as criteria or instances, the expert must make more pairwise comparisons to establish preferences. However, once these preferences are established, applying the results to different configurations has minimal computational impact. To mitigate these limitations, future work could explore partial weighting strategies, where the expert provides only a subset of preferences and the system extrapolates the remaining ones. Additionally, incorporating automated preference learning techniques, such as leveraging historical data or machine learning models, could reduce the dependency on manual input and enhance the objectivity and scalability of the framework.

Another practical implication is the reduction in time and resources spent on the configuration process. By automating prioritisation and providing clear, accountable results, the framework helps organisations streamline product development cycles and improve collaboration between stakeholders. This promotes better alignment with strategic goals and facilitates the delivery of high quality, customised products.

A comparative analysis of our approach with other prioritisation methods in the literature shows that the proposed integration of decision support mechanisms is distinctive in its ability to resolve discrepancies and facilitate informed decision-making. This feature is particularly relevant in scenarios where configuration options are closely ranked in priority and require detailed investigation to determine the optimal choice.

In addition to SPLs, the framework can be adapted to other decision-making environments where complex configurations are essential. This includes adaptive systems, personalised software environments, and even hardware-software co-design scenarios, underscoring the versatility of the approach.

This discussion highlights the potential and applicability of the proposed methodology in a range of domains where configuration prioritisation is a key consideration. It provides a structured yet flexible approach that can be adapted to different expert needs and decision contexts.

## Related work

Configuration prioritisation is an open problem in the SPL community. However, the concept of prioritisation has already been discussed in the field of feature modelling, although, to the best of our knowledge, not for the same purposes as our work. Related work has been distributed according to the following topics:

1. **Optimisation and prioritisation of the configurations of the feature models.** Incorporating user preferences into feature selection in SPL is not a new problem. Saber, Bendechache & Ventresque (2021) presents a solution based on a genetic algorithm to find the optimal configuration according to a multi-objective function. However, the definition of the criteria for sorting the configurations is not supported.
  Contributions such as Bertsimas & Mišić (2019) facilitate the selection of product configuration to maximise profit based on preferences, which is known as the product line design (PLD) problem.
  The approach in Guo et al. (2019) proposes a hybrid multi-objective optimisation algorithm using the indicator-based evolutionary algorithm (IBEA) with satisfiability modulo theories (SMT) to solve.
  Furthermore, Nguyen et al. (2019) provide a prioritisation technique for configurable systems by ranking the configurations according to their total number of potential bugs.
  The contribution of Sánchez & Segura (2017) presents SmarTest, a test prioritisation tool to speed up the detection of bugs in the Drupal web content management framework. It allows the execution of tests based on the real data of the Drupal product undergoing evaluation and the selection of the tests to be executed in the established order.
  In the approach of Sánchez, Segura & Ruiz-Cortés (2014), the applicability of test case prioritisation techniques to the evaluation of Software Product Lines (SPL) is explored. The article proposes five different prioritisation criteria based on common feature model metrics and compares their effectiveness in increasing the early fault detection rate, which measures how quickly faults are detected.
  Recent studies in multi-objective optimisation for SPLs, such as Jamil et al. (2023), Marghny et al. (2022), Abbas, Siddiqui & Lee (2016), highlight the increasing use of sophisticated heuristics and hybrid techniques to improve configuration selection. However, these works primarily focus on optimising performance rather than incorporating structured multi-criteria decision-making approaches as proposed in our work.
2. **Application of multi-criteria decision-making to software product line.** MCDM is not new in the context of SPLs, the approach in Thurimella & Ramaswamy (2012) proposed a hybrid quantitative and qualitative method based on Issue-based Variability Management (IVM). However, the proposal does not sort every possible configuration obtained, it is focussed on assigning prioritisation in each variation point, but not sorting all the products obtained.
  Beyond the SPL domain, decision-making frameworks have been widely applied across numerous fields, reflecting their adaptability and continuous evolution. Recent studies highlight their use in diverse areas such as renewable energy systems (Mukelabai, Barbour & Blanchard, 2024), transportation planning (Kriswardhana et al., 2025), recommender systems (Anwar et al., 2025), and AI-based managerial decision support (Marocco, Barbieri & Talamo, 2024). These works illustrate how decision-making approaches, including MCDM techniques, help structure complex decisions, balance multiple criteria, and enhance strategic planning. Despite this broad applicability, no prior research has systematically integrated multiple MCDM methods to resolve ranking discrepancies in feature model configurations, making our contribution distinct within the SPL context.
  Previous works have explored the use of decision-making frameworks in SPLs (Cao et al., 2024; Farshidi et al., 2018; Zaidan et al., 2015), but they do not integrate multiple MCDM methods to resolve discrepancies among rankings.
3. **Comparative analysis of the different rankings obtained through MCDM.** In the contribution by Shekhovtsov & Sałabun (2020), the ranking similarity obtained by VIKOR and TOPSIS is analysed using three ranking similarity coefficients. The comparison results of the conducted simulations are represented as boxplots.
  Yadav, Goraya & Singh (2021) evaluates AHP, PROMETHEE II, TOPSIS, and VIKOR in cloud environments for service selection. The comparative analysis includes differences and similarities in rankings, application-specific analysis, sensitivity analysis, and ranking overhead.
  Although PROMETHEE and ELECTRE are well-known MCDM techniques (Azhar, Radzi & Ahmad, 2021; Behzadian et al., 2010), they have not been included in our study as they were not among the selected methods for this work. Nevertheless, future studies may explore their integration into feature model prioritisation.
4. **Integration of fuzzy logic in MCDM methods.** Fuzzy-based MCDM approaches have been gaining relevance in recent years to handle uncertainty in decision-making scenarios. In particular, extensions like hesitant fuzzy sets, bipolar fuzzy sets, and linear Diophantine fuzzy sets (Aslam et al., 2024; Mahmood et al., 2022; Panpho & Yiarayong, 2023 Kahraman, Cebeci & Ruan, 2004) have been applied to complex prioritisation problems. While our approach does not incorporate these methods, future extensions could explore their applicability in handling ambiguous or conflicting expert preferences.

Related to the solutions that support the automatic reasoning of configurations, there are several tools, such as Flama (https://flamapy.github.io/), FeatureIDE (https://www.featureide.de/), SPL-Conqueror (https://www.se.cs.uni-saarland.de/projects/splconqueror/), SPLOT (http://splot-research.org/) and FAMILIAR (https://familiar-project.github.io/). Unfortunately, none of them provide the possibility to define a prioritisation criterion to obtain the possible configurations based on it, let alone to decide between different ranking lists of configurations.

## Conclusions and future work

This study has introduced a methodology for the prioritisation of configurations obtained from feature models (FMs) using MCDM techniques, specifically AHP, TOPSIS, and VIKOR. The increasing complexity of modern SPLs, where feature models can generate thousands of valid configurations, necessitates the selection of the most suitable configuration based on user preferences, which is a critical challenge.

The principal contribution of this work lies in the implementation of MCDM methodologies for configuration prioritisation, in addition to the introduction of a systematic approach for the management of discrepancies between rankings generated by disparate techniques. The proposed framework offers a structured decision-support mechanism that allows experts to analyse trade-offs between configurations and make informed selections. While MCDM methods have been individually applied in other decision-making contexts, the present study demonstrates the benefits of integrating multiple techniques to enhance decision reliability in feature-based configuration problems.

Furthermore, we have implemented this approach in an available framework, allowing practitioners to apply our methodology in real-world application domains. The methodology has been validated with two complex feature models published in the literature, demonstrating its practical feasibility and robustness. The results show that different MCDM methods, despite being based on the same expert-defined preferences, can lead to different rankings, reinforcing the importance of an informed decision-making phase to reconcile discrepancies.

Despite the achievements of this work, several directions could be explored in future research. For example: (1) expanding the methodology through the incorporation of additional MCDM techniques or hybrid approaches, to enhance ranking consistency and accuracy; (2) developing enhanced visualisation techniques to facilitate more effective communication of discrepancies between rankings, thereby enabling users to gain a more profound understanding of the prioritisation results; (3) testing the framework in broader application domains, such as IoT systems or cloud-based environments, to assess its scalability and adaptability to different contexts; (4) exploring advanced automation strategies, such as machine learning-based preference learning, to reduce the manual effort required in expert-driven weighting processes; (5) the incorporation of more advanced mathematical frameworks for handling uncertainty in decision-making, such as hesitant bipolar complex fuzzy sets (Aslam et al., 2024; Mahmood et al., 2022). These approaches have the potential to facilitate a more detailed representation of expert preferences, particularly in cases where decision-makers express reluctance or divergent priorities. The integration of such models with MCDM methods has the potential to yield further insights and enhance the robustness of prioritisation in complex feature models; and (6) conducting empirical evaluations with large-scale feature models and real-world datasets to assess the efficiency and usability of the framework in practical industrial scenarios. This would involve benchmarking the framework against other prioritisation tools and decision-making approaches to provide a quantitative assessment of its advantages and limitations.

By addressing these challenges, future work can further enhance the applicability and impact of the proposed methodology, ensuring that prioritisation in feature-based configuration remains both rigorous and user-friendly.

## Additional Information and Declarations

### Data Availability

The following information was supplied regarding data availability:

The AMADEUS framework code is available at Zenodo: Ángel J. Varela Vaca. (2025). ajvarela/amadeus-exploit-peerj25: Informed decision-making in prioritising product variants (v1.1). Zenodo. https://doi.org/10.5281/zenodo.14870223.
